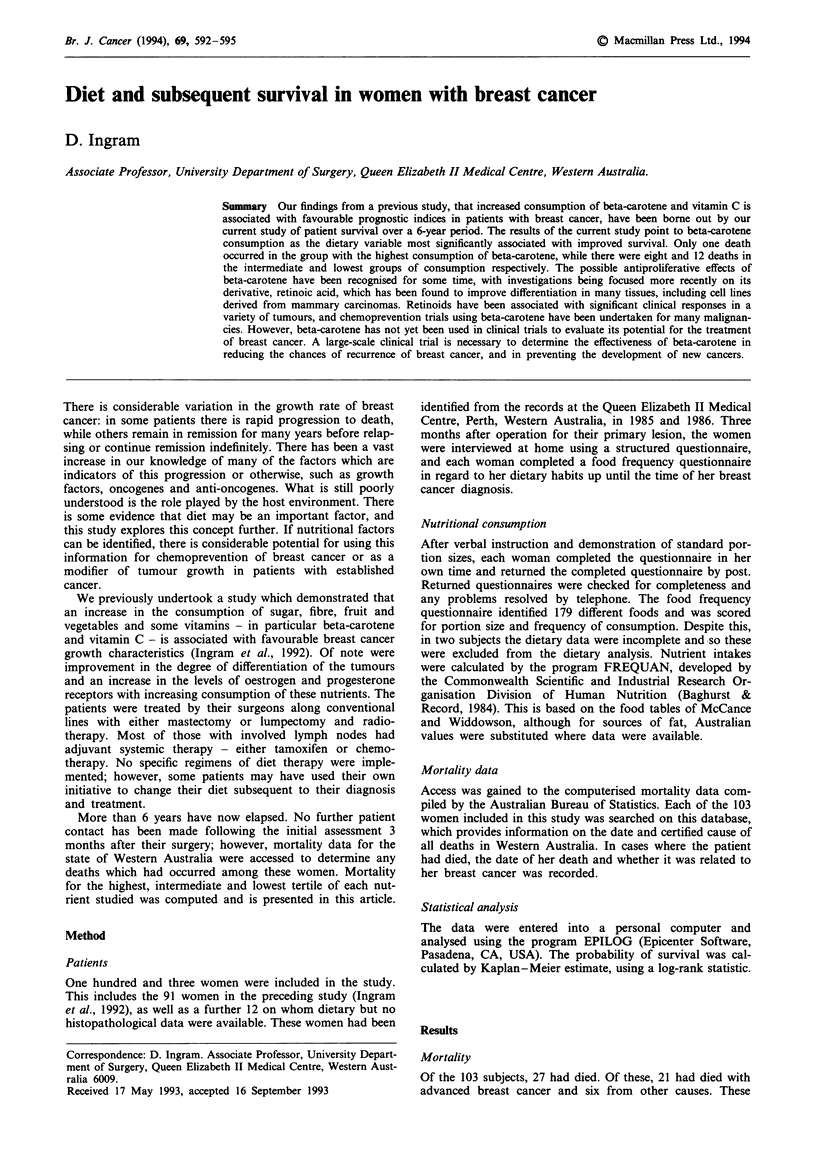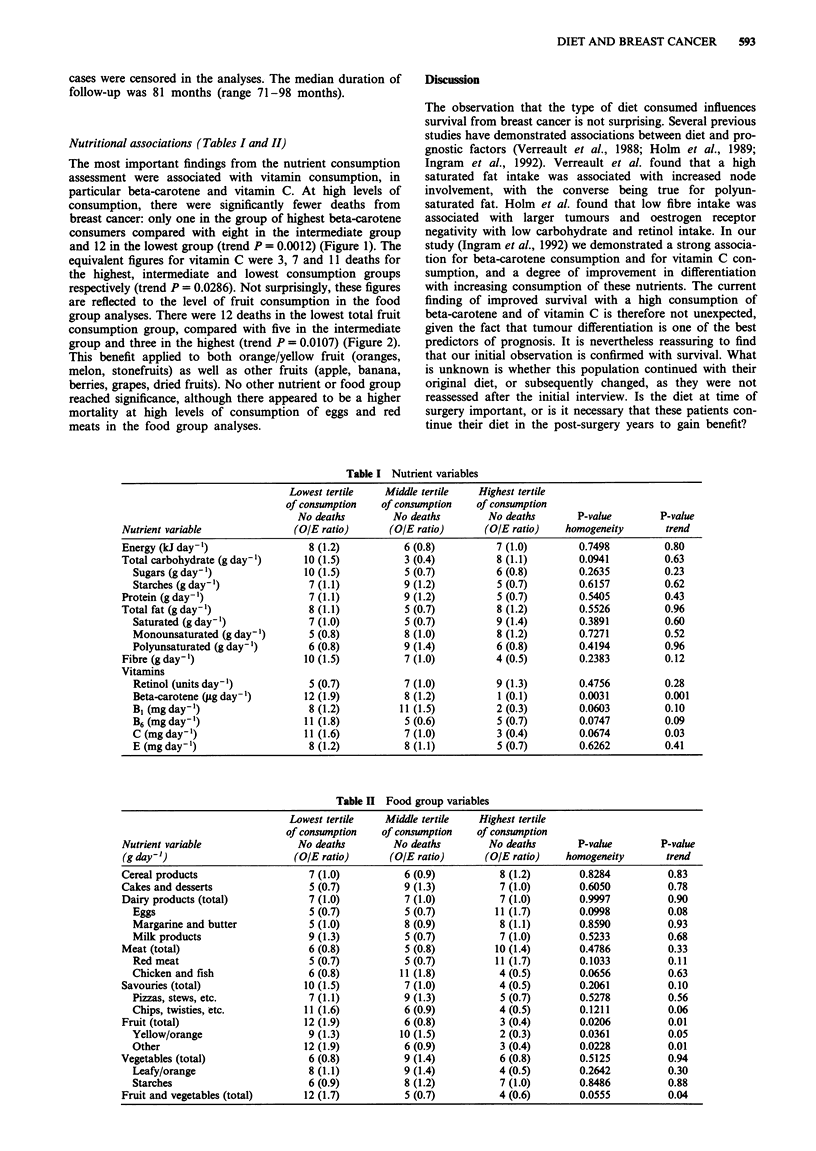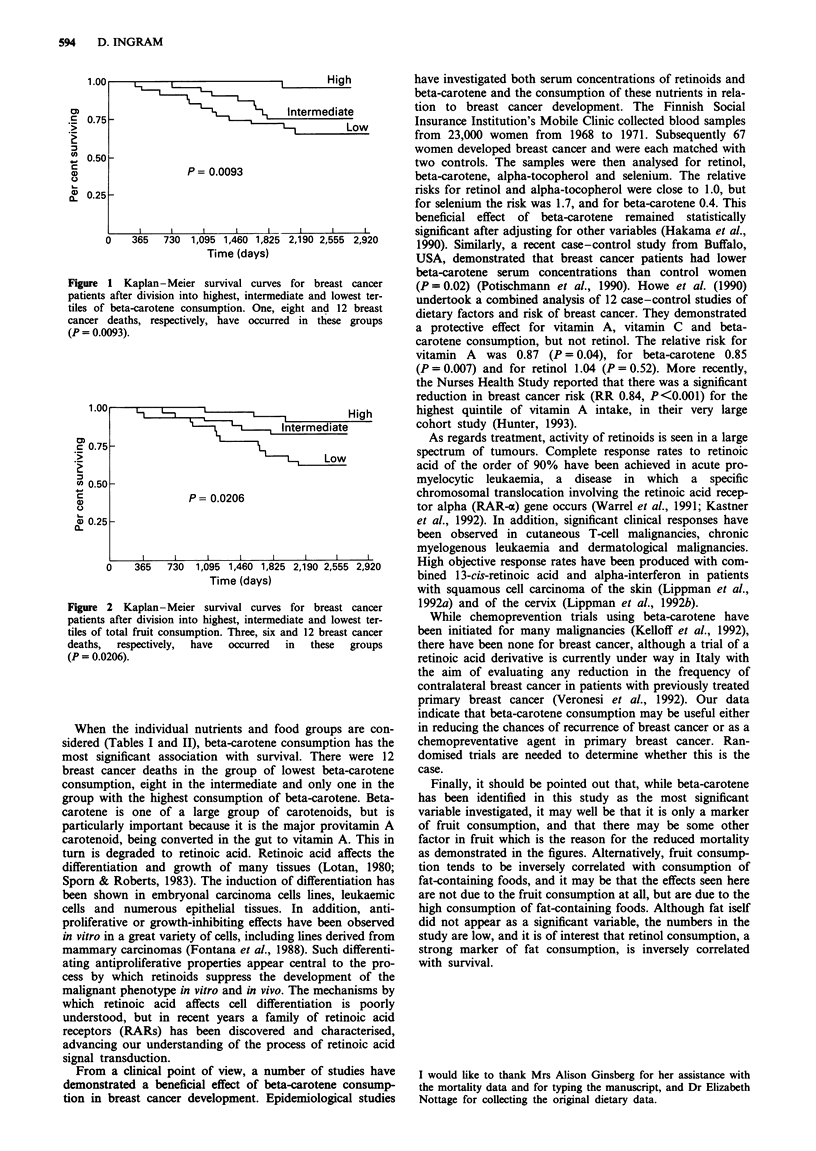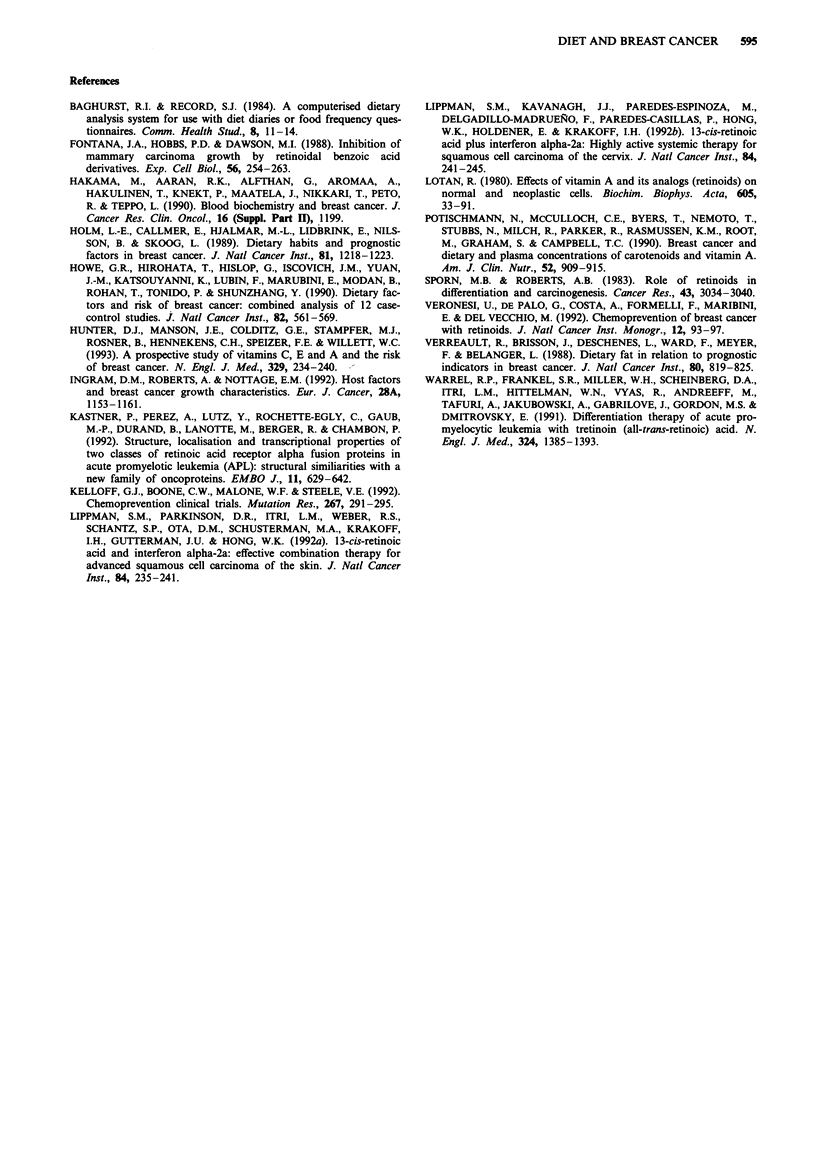# Diet and subsequent survival in women with breast cancer.

**DOI:** 10.1038/bjc.1994.108

**Published:** 1994-03

**Authors:** D. Ingram

**Affiliations:** University Department of Surgery, Queen Elizabeth II Medical Centre, Western Australia.

## Abstract

Our findings from a previous study, that increased consumption of beta-carotene and vitamin C is associated with favourable prognostic indices in patients with breast cancer, have been borne out by our current study of patient survival over a 6-year period. The results of the current study point to beta-carotene consumption as the dietary variable most significantly associated with improved survival. Only one death occurred in the group with the highest consumption of beta-carotene, while there were eight and 12 deaths in the intermediate and lowest groups of consumption respectively. The possible antiproliferative effects of beta-carotene have been recognised for some time, with investigations being focused more recently on its derivative, retinoic acid, which has been found to improve differentiation in many tissues, including cell lines derived from mammary carcinomas. Retinoids have been associated with significant clinical responses in a variety of tumours, and chemoprevention trials using beta-carotene have been undertaken for many malignancies. However, beta-carotene has not yet been used in clinical trials to evaluate its potential for the treatment of breast cancer. A large-scale clinical trial is necessary to determine the effectiveness of beta-carotene in reducing the chances of recurrence of breast cancer, and in preventing the development of new cancers.


*Br. J. Cancer* (1994), **69**, 592–595 © Macmillan Press Ltd., 1994

### Diet and subsequent survival in women with breast cancer

D. Ingram

*Associate Professor, University Department of Surgery, Queen Elizabeth II Medical Centre, Western Australia.*

**Summary** Our findings from a previous study, that increased consumption of beta-carotene and vitamin C is associated with favourable prognostic indices in patients with breast cancer, have been borne out by our current study of patient survival over a 6-year period. The results of the current study point to beta-carotene consumption as the dietary variable most significantly associated with improved survival. Only one death occurred in the group with the highest consumption of beta-carotene, while there were eight and 12 deaths in the intermediate and lowest groups of consumption respectively. The possible antiproliferative effects of beta-carotene have been recognised for some time, with investigations being focused more recently on its derivative, retinoic acid, which has been found to improve differentiation in many tissues, including cell lines derived from mammary carcinomas. Retinoids have been associated with significant clinical responses in a variety of tumours, and chemoprevention trials using beta-carotene have been undertaken for many malignancies. However, beta-carotene has not yet been used in clinical trials to evaluate its potential for the treatment of breast cancer. A large-scale clinical trial is necessary to determine the effectiveness of beta-carotene in reducing the chances of recurrence of breast cancer, and in preventing the development of new cancers.

There is considerable variation in the growth rate of breast cancer: in some patients there is rapid progression to death, while others remain in remission for many years before relapsing or continue remission indefinitely. There has been a vast increase in our knowledge of many of the factors which are indicators of this progression or otherwise, such as growth factors, oncogenes and anti-oncogenes. What is still poorly understood is the role played by the host environment. There is some evidence that diet may be an important factor, and this study explores this concept further. If nutritional factors can be identified, there is considerable potential for using this information for chemoprevention of breast cancer or as a modifier of tumour growth in patients with established cancer.

We previously undertook a study which demonstrated that an increase in the consumption of sugar, fibre, fruit and vegetables and some vitamins – in particular beta-carotene and vitamin C – is associated with favourable breast cancer growth characteristics (Ingram *et al.*, 1992). Of note were improvement in the degree of differentiation of the tumours and an increase in the levels of oestrogen and progesterone receptors with increasing consumption of these nutrients. The patients were treated by their surgeons along conventional lines with either mastectomy or lumpectomy and radiotherapy. Most of those with involved lymph nodes had adjuvant systemic therapy – either tamoxifen or chemotherapy. No specific regimens of diet therapy were implemented; however, some patients may have used their own initiative to change their diet subsequent to their diagnosis and treatment.

More than 6 years have now elapsed. No further patient contact has been made following the initial assessment 3 months after their surgery; however, mortality data for the state of Western Australia were accessed to determine any deaths which had occurred among these women. Mortality for the highest, intermediate and lowest tertile of each nutrient studied was computed and is presented in this article.

### Method

### Patients

One hundred and three women were included in the study. This includes the 91 women in the preceding study (Ingram *et al.*, 1992), as well as a further 12 on whom dietary but no histopathological data were available. These women had been identified from the records at the Queen Elizabeth II Medical Centre, Perth, Western Australia, in 1985 and 1986. Three months after operation for their primary lesion, the women were interviewed at home using a structured questionnaire, and each woman completed a food frequency questionnaire in regard to her dietary habits up until the time of her breast cancer diagnosis.

Correspondence: D. Ingram. Associate Professor, University Department of Surgery, Queen Elizabeth II Medical Centre, Western Australia 6009.
Received 17 May 1993, accepted 16 September 1993

### Nutritional consumption

After verbal instruction and demonstration of standard portion sizes, each woman completed the questionnaire in her own time and returned the completed questionnaire by post. Returned questionnaires were checked for completeness and any problems resolved by telephone. The food frequency questionnaire identified 179 different foods and was scored for portion size and frequency of consumption. Despite this, in two subjects the dietary data were incomplete and so these were excluded from the dietary analysis. Nutrient intakes were calculated by the program FREQUAN, developed by the Commonwealth Scientific and Industrial Research Organisation Division of Human Nutrition (Baghurst & Record, 1984). This is based on the food tables of McCance and Widdowson, although for sources of fat, Australian values were substituted where data were available.

### Mortality data

Access was gained to the computerised mortality data compiled by the Australian Bureau of Statistics. Each of the 103 women included in this study was searched on this database, which provides information on the date and certified cause of all deaths in Western Australia. In cases where the patient had died, the date of her death and whether it was related to her breast cancer was recorded.

### Statistical analysis

The data were entered into a personal computer and analysed using the program EPILOG (Epicenter Software, Pasadena, CA, USA). The probability of survival was calculated by Kaplan–Meier estimate, using a log-rank statistic.

### Results

### Mortality

Of the 103 subjects, 27 had died. Of these, 21 had died with advanced breast cancer and six from other causes. These cases were censored in the analyses. The median duration of follow-up was 81 months (range 71–98 months).

*Nutritional associations (Tables I and II)*

The most important findings from the nutrient consumption assessment were associated with vitamin consumption, in particular beta-carotene and vitamin C. At high levels of consumption, there were significantly fewer deaths from breast cancer: only one in the group of highest beta-carotene consumers compared with eight in the intermediate group and 12 in the lowest group (trend $P = 0.0012$) (Figure 1). The equivalent figures for vitamin C were 3, 7 and 11 deaths for the highest, intermediate and lowest consumption groups respectively (trend $P = 0.0286$). Not surprisingly, these figures are reflected to the level of fruit consumption in the food group analyses. There were 12 deaths in the lowest total fruit consumption group, compared with five in the intermediate group and three in the highest (trend $P = 0.0107$) (Figure 2). This benefit applied to both orange/yellow fruit (oranges, melon, stonefruits) as well as other fruits (apple, banana, berries, grapes, dried fruits). No other nutrient or food group reached significance, although there appeared to be a higher mortality at high levels of consumption of eggs and red meats in the food group analyses.

### Discussion

The observation that the type of diet consumed influences survival from breast cancer is not surprising. Several previous studies have demonstrated associations between diet and prognostic factors (Verreault *et al.*, 1988; Holm *et al.*, 1989; Ingram *et al.*, 1992). Verreault *et al.* found that a high saturated fat intake was associated with increased node involvement, with the converse being true for polyunsaturated fat. Holm *et al.* found that low fibre intake was associated with larger tumours and oestrogen receptor negativity with low carbohydrate and retinol intake. In our study (Ingram *et al.*, 1992) we demonstrated a strong association for beta-carotene consumption and for vitamin C consumption, and a degree of improvement in differentiation with increasing consumption of these nutrients. The current finding of improved survival with a high consumption of beta-carotene and of vitamin C is therefore not unexpected, given the fact that tumour differentiation is one of the best predictors of prognosis. It is nevertheless reassuring to find that our initial observation is confirmed with survival. What is unknown is whether this population continued with their original diet, or subsequently changed, as they were not reassessed after the initial interview. Is the diet at time of surgery important, or is it necessary that these patients continue their diet in the post-surgery years to gain benefit?

**Table I** Nutrient variables

| *Nutrient variable* | *Lowest tertile of consumption No deaths (O/E ratio)* | *Middle tertile of consumption No deaths (O/E ratio)* | *Highest tertile of consumption No deaths (O/E ratio)* | P-*value homogeneity* | P-*value trend* |
|---|---|---|---|---|---|
| Energy (kJ day$^{-1}$) | 8 (1.2) | 6 (0.8) | 7 (1.0) | 0.7498 | 0.80 |
| Total carbohydrate (g day$^{-1}$) | 10 (1.5) | 3 (0.4) | 8 (1.1) | 0.0941 | 0.63 |
| Sugars (g day$^{-1}$) | 10 (1.5) | 5 (0.7) | 6 (0.8) | 0.2635 | 0.23 |
| Starches (g day$^{-1}$) | 7 (1.1) | 9 (1.2) | 5 (0.7) | 0.6157 | 0.62 |
| Protein (g day$^{-1}$) | 7 (1.1) | 9 (1.2) | 5 (0.7) | 0.5405 | 0.43 |
| Total fat (g day$^{-1}$) | 8 (1.1) | 5 (0.7) | 8 (1.2) | 0.5526 | 0.96 |
| Saturated (g day$^{-1}$) | 7 (1.0) | 5 (0.7) | 9 (1.4) | 0.3891 | 0.60 |
| Monounsaturated (g day$^{-1}$) | 5 (0.8) | 8 (1.0) | 8 (1.2) | 0.7271 | 0.52 |
| Polyunsaturated (g day$^{-1}$) | 6 (0.8) | 9 (1.4) | 6 (0.8) | 0.4194 | 0.96 |
| Fibre (g day$^{-1}$) | 10 (1.5) | 7 (1.0) | 4 (0.5) | 0.2383 | 0.12 |
| Vitamins | | | | | |
| Retinol (units day$^{-1}$) | 5 (0.7) | 7 (1.0) | 9 (1.3) | 0.4756 | 0.28 |
| Beta-carotene (μg day$^{-1}$) | 12 (1.9) | 8 (1.2) | 1 (0.1) | 0.0031 | 0.001 |
| $B_1$ (mg day$^{-1}$) | 8 (1.2) | 11 (1.5) | 2 (0.3) | 0.0603 | 0.10 |
| $B_6$ (mg day$^{-1}$) | 11 (1.8) | 5 (0.6) | 5 (0.7) | 0.0747 | 0.09 |
| C (mg day$^{-1}$) | 11 (1.6) | 7 (1.0) | 3 (0.4) | 0.0674 | 0.03 |
| E (mg day$^{-1}$) | 8 (1.2) | 8 (1.1) | 5 (0.7) | 0.6262 | 0.41 |

**Table II** Food group variables

| *Nutrient variable (g day$^{-1}$)* | *Lowest tertile of consumption No deaths (O/E ratio)* | *Middle tertile of consumption No deaths (O/E ratio)* | *Highest tertile of consumption No deaths (O/E ratio)* | P-*value homogeneity* | P-*value trend* |
|---|---|---|---|---|---|
| Cereal products | 7 (1.0) | 6 (0.9) | 8 (1.2) | 0.8284 | 0.83 |
| Cakes and desserts | 5 (0.7) | 9 (1.3) | 7 (1.0) | 0.6050 | 0.78 |
| Dairy products (total) | 7 (1.0) | 7 (1.0) | 7 (1.0) | 0.9997 | 0.90 |
| Eggs | 5 (0.7) | 5 (0.7) | 11 (1.7) | 0.0998 | 0.08 |
| Margarine and butter | 5 (1.0) | 8 (0.9) | 8 (1.1) | 0.8590 | 0.93 |
| Milk products | 9 (1.3) | 5 (0.7) | 7 (1.0) | 0.5233 | 0.68 |
| Meat (total) | 6 (0.8) | 5 (0.8) | 10 (1.4) | 0.4786 | 0.33 |
| Red meat | 5 (0.7) | 5 (0.7) | 11 (1.7) | 0.1033 | 0.11 |
| Chicken and fish | 6 (0.8) | 11 (1.8) | 4 (0.5) | 0.0656 | 0.63 |
| Savouries (total) | 10 (1.5) | 7 (1.0) | 4 (0.5) | 0.2061 | 0.10 |
| Pizzas, stews, etc. | 7 (1.1) | 9 (1.3) | 5 (0.7) | 0.5278 | 0.56 |
| Chips, twisties, etc. | 11 (1.6) | 6 (0.9) | 4 (0.5) | 0.1211 | 0.06 |
| Fruit (total) | 12 (1.9) | 6 (0.8) | 3 (0.4) | 0.0206 | 0.01 |
| Yellow/orange | 9 (1.3) | 10 (1.5) | 2 (0.3) | 0.0361 | 0.05 |
| Other | 12 (1.9) | 6 (0.9) | 3 (0.4) | 0.0228 | 0.01 |
| Vegetables (total) | 6 (0.8) | 9 (1.4) | 6 (0.8) | 0.5125 | 0.94 |
| Leafy/orange | 8 (1.1) | 9 (1.4) | 4 (0.5) | 0.2642 | 0.30 |
| Starches | 6 (0.9) | 8 (1.2) | 7 (1.0) | 0.8486 | 0.88 |
| Fruit and vegetables (total) | 12 (1.7) | 5 (0.7) | 4 (0.6) | 0.0555 | 0.04 |

**Figure 1** Kaplan–Meier survival curves for breast cancer patients after division into highest, intermediate and lowest tertiles of beta-carotene consumption. One, eight and 12 breast cancer deaths, respectively, have occurred in these groups ($P = 0.0093$).

**Figure 2** Kaplan–Meier survival curves for breast cancer patients after division into highest, intermediate and lowest tertiles of total fruit consumption. Three, six and 12 breast cancer deaths, respectively, have occurred in these groups ($P = 0.0206$).

When the individual nutrients and food groups are considered (Tables I and II), beta-carotene consumption has the most significant association with survival. There were 12 breast cancer deaths in the group of lowest beta-carotene consumption, eight in the intermediate and only one in the group with the highest consumption of beta-carotene. Beta-carotene is one of a large group of carotenoids, but is particularly important because it is the major provitamin A carotenoid, being converted in the gut to vitamin A. This in turn is degraded to retinoic acid. Retinoic acid affects the differentiation and growth of many tissues (Lotan, 1980; Sporn & Roberts, 1983). The induction of differentiation has been shown in embryonal carcinoma cells lines, leukaemic cells and numerous epithelial tissues. In addition, anti-proliferative or growth-inhibiting effects have been observed *in vitro* in a great variety of cells, including lines derived from mammary carcinomas (Fontana *et al.*, 1988). Such differentiating antiproliferative properties appear central to the process by which retinoids suppress the development of the malignant phenotype *in vitro* and *in vivo*. The mechanisms by which retinoic acid affects cell differentiation is poorly understood, but in recent years a family of retinoic acid receptors (RARs) has been discovered and characterised, advancing our understanding of the process of retinoic acid signal transduction.

From a clinical point of view, a number of studies have demonstrated a beneficial effect of beta-carotene consumption in breast cancer development. Epidemiological studies have investigated both serum concentrations of retinoids and beta-carotene and the consumption of these nutrients in relation to breast cancer development. The Finnish Social Insurance Institution's Mobile Clinic collected blood samples from 23,000 women from 1968 to 1971. Subsequently 67 women developed breast cancer and were each matched with two controls. The samples were then analysed for retinol, beta-carotene, alpha-tocopherol and selenium. The relative risks for retinol and alpha-tocopherol were close to 1.0, but for selenium the risk was 1.7, and for beta-carotene 0.4. This beneficial effect of beta-carotene remained statistically significant after adjusting for other variables (Hakama *et al.*, 1990). Similarly, a recent case–control study from Buffalo, USA, demonstrated that breast cancer patients had lower beta-carotene serum concentrations than control women ($P = 0.02$) (Potischmann *et al.*, 1990). Howe *et al.* (1990) undertook a combined analysis of 12 case–control studies of dietary factors and risk of breast cancer. They demonstrated a protective effect for vitamin A, vitamin C and beta-carotene consumption, but not retinol. The relative risk for vitamin A was 0.87 ($P = 0.04$), for beta-carotene 0.85 ($P = 0.007$) and for retinol 1.04 ($P = 0.52$). More recently, the Nurses Health Study reported that there was a significant reduction in breast cancer risk (RR 0.84, $P < 0.001$) for the highest quintile of vitamin A intake, in their very large cohort study (Hunter, 1993).

As regards treatment, activity of retinoids is seen in a large spectrum of tumours. Complete response rates to retinoic acid of the order of 90% have been achieved in acute promyelocytic leukaemia, a disease in which a specific chromosomal translocation involving the retinoic acid receptor alpha (RAR-α) gene occurs (Warrel *et al.*, 1991; Kastner *et al.*, 1992). In addition, significant clinical responses have been observed in cutaneous T-cell malignancies, chronic myelogenous leukaemia and dermatological malignancies. High objective response rates have been produced with combined 13-*cis*-retinoic acid and alpha-interferon in patients with squamous cell carcinoma of the skin (Lippman *et al.*, 1992*a*) and of the cervix (Lippman *et al.*, 1992*b*).

While chemoprevention trials using beta-carotene have been initiated for many malignancies (Kelloff *et al.*, 1992), there have been none for breast cancer, although a trial of a retinoic acid derivative is currently under way in Italy with the aim of evaluating any reduction in the frequency of contralateral breast cancer in patients with previously treated primary breast cancer (Veronesi *et al.*, 1992). Our data indicate that beta-carotene consumption may be useful either in reducing the chances of recurrence of breast cancer or as a chemopreventative agent in primary breast cancer. Randomised trials are needed to determine whether this is the case.

Finally, it should be pointed out that, while beta-carotene has been identified in this study as the most significant variable investigated, it may well be that it is only a marker of fruit consumption, and that there may be some other factor in fruit which is the reason for the reduced mortality as demonstrated in the figures. Alternatively, fruit consumption tends to be inversely correlated with consumption of fat-containing foods, and it may be that the effects seen here are not due to the fruit consumption at all, but are due to the high consumption of fat-containing foods. Although fat iself did not appear as a significant variable, the numbers in the study are low, and it is of interest that retinol consumption, a strong marker of fat consumption, is inversely correlated with survival.

I would like to thank Mrs Alison Ginsberg for her assistance with the mortality data and for typing the manuscript, and Dr Elizabeth Nottage for collecting the original dietary data.

### References

BAGHURST, R.I. & RECORD, S.J. (1984). A computerised dietary analysis system for use with diet diaries or food frequency questionnaires. *Comm. Health Stud.*, **8**, 11–14.

FONTANA, J.A., HOBBS, P.D. & DAWSON, M.I. (1988). Inhibition of mammary carcinoma growth by retinoidal benzoic acid derivatives. *Exp. Cell Biol.*, **56**, 254–263.

HAKAMA, M., AARAN, R.K., ALFTHAN, G., AROMAA, A., HAKULINEN, T., KNEKT, P., MAATELA, J., NIKKARI, T., PETO, R. & TEPPO, L. (1990). Blood biochemistry and breast cancer. *J. Cancer Res. Clin. Oncol.*, **16 (Suppl. Part II)**, 1199.

HOLM, L.-E., CALLMER, E., HJALMAR, M.-L., LIDBRINK, E., NILSSON, B. & SKOOG, L. (1989). Dietary habits and prognostic factors in breast cancer. *J. Natl Cancer Inst.*, **81**, 1218–1223.

HOWE, G.R., HIROHATA, T., HISLOP, G., ISCOVICH, J.M., YUAN, J.-M., KATSOUYANNI, K., LUBIN, F., MARUBINI, E., MODAN, B., ROHAN, T., TONIDO, P. & SHUNZHANG, Y. (1990). Dietary factors and risk of breast cancer: combined analysis of 12 case-control studies. *J. Natl Cancer Inst.*, **82**, 561–569.

HUNTER, D.J., MANSON, J.E., COLDITZ, G.E., STAMPFER, M.J., ROSNER, B., HENNEKENS, C.H., SPEIZER, F.E. & WILLETT, W.C. (1993). A prospective study of vitamins C, E and A and the risk of breast cancer. *N. Engl. J. Med.*, **329**, 234–240.

INGRAM, D.M., ROBERTS, A. & NOTTAGE, E.M. (1992). Host factors and breast cancer growth characteristics. *Eur. J. Cancer*, **28A**, 1153–1161.

KASTNER, P., PEREZ, A., LUTZ, Y., ROCHETTE-EGLY, C., GAUB, M.-P., DURAND, B., LANOTTE, M., BERGER, R. & CHAMBON, P. (1992). Structure, localisation and transcriptional properties of two classes of retinoic acid receptor alpha fusion proteins in acute promyelotic leukemia (APL): structural similiarities with a new family of oncoproteins. *EMBO J.*, **11**, 629–642.

KELLOFF, G.J., BOONE, C.W., MALONE, W.F. & STEELE, V.E. (1992). Chemoprevention clinical trials. *Mutation Res.*, **267**, 291–295.

LIPPMAN, S.M., PARKINSON, D.R., ITRI, L.M., WEBER, R.S., SCHANTZ, S.P., OTA, D.M., SCHUSTERMAN, M.A., KRAKOFF, I.H., GUTTERMAN, J.U. & HONG, W.K. (1992*a*). 13-*cis*-retinoic acid and interferon alpha-2a: effective combination therapy for advanced squamous cell carcinoma of the skin. *J. Natl Cancer Inst.*, **84**, 235–241.

LIPPMAN, S.M., KAVANAGH, J.J., PAREDES-ESPINOZA, M., DELGADILLO-MADRUEÑO, F., PAREDES-CASILLAS, P., HONG, W.K., HOLDENER, E. & KRAKOFF, I.H. (1992*b*). 13-*cis*-retinoic acid plus interferon alpha-2a: Highly active systemic therapy for squamous cell carcinoma of the cervix. *J. Natl Cancer Inst.*, **84**, 241–245.

LOTAN, R. (1980). Effects of vitamin A and its analogs (retinoids) on normal and neoplastic cells. *Biochim. Biophys. Acta*, **605**, 33–91.

POTISCHMANN, N., MCCULLOCH, C.E., BYERS, T., NEMOTO, T., STUBBS, N., MILCH, R., PARKER, R., RASMUSSEN, K.M., ROOT, M., GRAHAM, S. & CAMPBELL, T.C. (1990). Breast cancer and dietary and plasma concentrations of carotenoids and vitamin A. *Am. J. Clin. Nutr.*, **52**, 909–915.

SPORN, M.B. & ROBERTS, A.B. (1983). Role of retinoids in differentiation and carcinogenesis. *Cancer Res.*, **43**, 3034–3040.

VERONESI, U., DE PALO, G., COSTA, A., FORMELLI, F., MARIBINI, E. & DEL VECCHIO, M. (1992). Chemoprevention of breast cancer with retinoids. *J. Natl Cancer Inst. Monogr.*, **12**, 93–97.

VERREAULT, R., BRISSON, J., DESCHENES, L., WARD, F., MEYER, F. & BELANGER, L. (1988). Dietary fat in relation to prognostic indicators in breast cancer. *J. Natl Cancer Inst.*, **80**, 819–825.

WARREL, R.P., FRANKEL, S.R., MILLER, W.H., SCHEINBERG, D.A., ITRI, L.M., HITTELMAN, W.N., VYAS, R., ANDREEFF, M., TAFURI, A., JAKUBOWSKI, A., GABRILOVE, J., GORDON, M.S. & DMITROVSKY, E. (1991). Differentiation therapy of acute promyelocytic leukemia with tretinoin (all-*trans*-retinoic) acid. *N. Engl. J. Med.*, **324**, 1385–1393.